# Dietary Fiber–Phenolic Milk Tablets Are Associated with Improved Lipid Profiles and Reduced Circulating HMGCR Levels in Hypercholesterolemic Subjects: An Open-Label Pre–Post Study

**DOI:** 10.3390/foods15122235

**Published:** 2026-06-21

**Authors:** Nut Palachai, Pontapan Polyiam, Sivamoke Dissook, Wasana Ko-iam, Pratoomporn Yingthongchai, Hechen Wang, Jurairat Khongrum

**Affiliations:** 1 Faculty of Medicine, Mahasarakham University, Maha Sarakham 44000, Thailand; nut.p@msu.ac.th; 2 Department of Medical Technology, Faculty of Allied Health Sciences, Nakhon Ratchasima College, Nakhon Ratchasima 30000, Thailand; pontapan@nmc.ac.th; 3 Department of Biochemistry, Faculty of Medicine, Chiang Mai University, Chiang Mai 50200, Thailand; sivamoke.dis@cmu.ac.th; 4 Research Unit, Department of Surgery, Faculty of Medicine, Chiang Mai University, Chiang Mai 50200, Thailand; wasana.ko-iam@cmu.ac.th; 5 Multidisciplinary Research Institute, Chiang Mai University, Chiang Mai 50200, Thailand; pratoomporn.y@cmu.ac.th; 6 Institute of Chinese Materia Medica, Shanghai University of Traditional Chinese Medicine, 1200 Cailun Road, Shanghai 201203, China; wang210723@163.com

**Keywords:** 3-Hydroxy-3-methylglutaryl-coenzyme A reductase (HMGCR), short-chain fatty acids, Apolipoprotein B100, dietary fiber

## Abstract

Modulation of cholesterol metabolism and reduction in serum cholesterol are key strategies for preventing cardiovascular diseases (CVDs). Functional foods enriched with dietary fiber and phytochemicals have attracted increasing attention for their potential health benefits. In this study, milk tablets containing kale and carrot (KC) were developed and preliminarily evaluated for their cholesterol-lowering potential. KC milk tablets were rich in dietary fiber, contained gallic acid, and exhibited antioxidant properties. They also supported the growth of *Lactobacillus casei* and *Bifidobacterium longum in vitro*, accompanied by increased SCFA production. In an open-label, pre–post exploratory study in hypercholesterolemic subjects, daily consumption for 6 weeks was associated with significantly increased HDL-C and reduced LDL-C levels. In addition, circulating ApoB100 and HMGCR levels were reduced, whereas ApoE and TNF-α remained unchanged. Therefore, these preliminary findings suggest that KC milk tablets may accomplish beneficial changes in lipid profiles and support the potential of dietary fiber–phenolic interactions with enhanced SCFA production which might modulate cholesterol metabolism. However, in further studies, randomized controlled trials are required to understand the precise underlying mechanism.

## 1. Introduction

Hypercholesterolemia, characterized by elevated circulating cholesterol levels, particularly low-density lipoprotein cholesterol (LDL-C), represents a well-established modifiable risk factor for atherosclerosis-related CVD development [1]. Consequently, dietary interventions aimed at maintaining cholesterol homeostasis have emerged as important strategies for atherosclerosis prevention [2]. Epidemiological studies consistently demonstrate an inverse dose–response relationship between vegetable consumption and the incidence of atherosclerosis and CVDs, partly through improvements in cholesterol metabolism, with meta-analyses indicating significant risk reductions associated with higher intakes [3,4]. The cholesterol-lowering properties of vegetables are attributed to their complex matrices of nutrition such as dietary fibers and phenolic compounds. Several studies have reported that the dietary fiber modulates cholesterol metabolism through multiple pathways such as bile acid sequestration, inhibition of cholesterol absorption, production of short-chain fatty acids (SCFAs) which suppress hepatic cholesterol synthesis via 3-Hydroxy-3-methylglutaryl-coenzyme A reductase (HMGCR) and modulation of gut microbiota composition [5,6,7]. Moreover, phytochemical constituents, particularly phenolic compounds in vegetables also contribute to cholesterol metabolism through antioxidant activity, anti-inflammatory effects, and direct modulation of lipid metabolic pathways [8,9]. The potential hypocholesterolemic effects of dietary fiber and phenolics play a crucial role in the development of functional foods, which are designed to provide health benefits beyond their basic nutritional value and have gained considerable attention in recent years.

Among these vegetables, carrot and kale are notable for their high nutritional value, particularly due to their abundant dietary fiber and diverse bioactive compounds. Recent studies have shown that carrot (*Daucus carota*) exhibits antioxidant, anti-inflammatory, cholesterol-regulating, and atheroprotective effects [10,11]. Kale (*Brassica oleracea* var. *acephala*) has been reported to possess various health-promoting properties, including antioxidant, anti-inflammatory, anticancer, cholesterol-lowering, and blood glucose–lowering effects, as well as beneficial effects on intestinal microflora [12,13].

Although carrot and kale have individually been suggested to be potentially associated with modulation of cholesterol metabolism, possibly contributing to a reduced risk of CVDs, the effects of their combined intake in a milk tablet formulation, including potential synergistic interaction between their dietary fiber and phenolic constituents, in modulating cholesterol metabolism in hypercholesterolemic individuals have not been reported. Therefore, the present study aimed to develop milk tablets containing kale and carrot (KC) as a functional food rich in dietary fiber and phenolic compounds, with potential prebiotic properties, including enhanced SCFA production. The effects of KC milk tablets on cholesterol metabolism, inflammatory cytokines, and lipid profiles were investigated, particularly in the context of hypercholesterolemic subjects. The present study may provide insights into their potential role as a dietary approach for supporting cholesterol metabolism.

## 2. Materials and Methods

### 2.1. Materials

The chemicals used in the present study were analytically pure and provided by Sigma-Aldrich (St. Louis, MO, USA) except as otherwise noted. Apolipoprotein E (ApoE) (Lot No. FN251009), apolipoprotein B100 (ApoB100) (Lot No. FN251009), 3-Hydroxy-3-methylglutaryl-coenzyme A reductase (HMGCR) (Lot No. FN251009), and tumor necrosis factor-alpha (TNF-α) (Lot No. FN251009) were quantified using enzyme-linked immunosorbent assay (ELISA) kits with paired antibodies purchased from Wuhan Fine Biotech Co., Ltd. (Wuhan, Hubei, China).

Gallic acid (Lot No. BCCN4299), Chlorogenic acid (Lot No. WXBD9324V), Vanillin (Lot No. BCBN9635V), *p*-Coumaric acid (Lot No. BCCL0049), Sinapic acid (Lot No. 00002957), Rutin (Lot No. BCBN0590V), Quercetin (Lot No. SLBD8415V), and Cinnamic acid (Lot No. MKBS4531V) were purchased from Sigma-Aldrich (St. Louis, MO, USA).

### 2.2. Preparation of Milk Tablets Enriched with Dietary Fiber and Phenolics from Kale and Carrot Powder

Kale leaves were obtained from Puk Doi OK Farm, Hot District, Chiang Mai Province, Thailand. Fully mature leaves were thoroughly washed, cut into small pieces, and freeze-dried to obtain kale powder. Carrots were collected from Phang District, Chiang Mai Province, Thailand. The roots were washed, cut into small pieces, and dried in a hot-air oven at 65 °C to obtain carrot powder. The combined kale and carrot powders were mixed with dry whole milk, non-dairy creamer, Khao Soi paste flavor, and other excipients to prepare KC milk tablets. Nutritional composition was analyzed by the Central Laboratory (Thailand) Co., Ltd., Chiang Mai Branch, which is accredited under ISO/IEC 17025:2017 [14], Don Kaeo, Mae Rim, Chiang Mai, Thailand.

### 2.3. Determination of Total Phenolic, Flavonoid Contents, and Antioxidant Activities

Total phenolic content, total flavonoid content, and antioxidant capacity of KC milk tablets were determined. Total phenolic content was measured using the Folin–Ciocalteu reagent and expressed as mg gallic acid equivalents per gram of dry weight (mg GAE/g DW) [15]. Total flavonoid content was determined by the aluminium chloride colorimetric method [16] and expressed as mg quercetin equivalents per gram of dry weight (mg QE/g DW). Antioxidant activities were evaluated using the 2,2′-diphenyl-1-picrylhydrazyl (DPPH) radical scavenging assay [17], the ferric reducing antioxidant power (FRAP) assay [18], and the ABTS radical cation scavenging assay [19]. Antioxidant activity was expressed as the EC_50_ value (mg/mL), defined as the effective concentration required to achieve 50% inhibition.

### 2.4. Quantification of Polyphenolic Compounds by High-Performance Liquid Chromatography (HPLC)

Polyphenolic compounds in KC milk tablets were identified and quantified using HPLC according to the method reported by Stoenescu et al. (2022) [20]. The identified compounds included gallic acid, chlorogenic acid, vanillin, *p*-coumaric acid, sinapic acid, rutin, quercetin, and cinnamic acid. In brief, the mobile phase consisted of aqueous acetic acid and methanol (MeOH). Gradient elution was performed according to a previously described method [20]. The flow rate was 0.8 mL/min, and the injection volume was 5 µL. Separation was carried out on an Agilent Eclipse XDB-C18 column (250 mm × 4.6 mm, 5 µm) at 30 °C. Polyphenolic compounds were detected at 278 nm. Identification was based on retention times and comparison with reference standards analyzed under the same conditions. Results were expressed as mg/g dry weight (DW) and presented as mean ± SD (*n* = 3).

### 2.5. In Vitro Evaluation of the Prebiotic Potential of KC Milk Tablets Using Probiotic Strains

The prebiotic potential of KC milk tablets was evaluated using two probiotic strains, *Lactobacillus casei* and *Bifidobacterium longum*, obtained from the Thailand Institute of Scientific and Technological Research (TISTR). The experimental procedures were conducted as follows. Rat feces were collected independently from three separate animals (*n* = 3), providing a standardized fermentation matrix containing endogenous nutrients that support probiotic growth. For basal fermentation preparation, 0.5 mL of 1× phosphate-buffered saline (PBS) was dispensed into test tubes containing 1 g of rat feces. The sample tubes were then sterilized by autoclaving for 15 min to eliminate endogenous microbial populations that could confound bacterial enumeration. The probiotic strains were prepared and adjusted to an optical density (OD_660_) of 0.5. A 500 µL aliquot of each standardized inoculum (*L. casei* or *B. longum*) was added to the fermentation tubes, while the control group remained uninoculated. Subsequently, 0.25 g of the sample was added to each tube. The mixtures were thoroughly homogenized using a vortex mixer (Scientific Industries, Inc., Bohemia, NY, USA).

After preparing serial dilutions of the samples, 0.1 mL aliquots of dilutions ranging from 10^−3^ to 10^−6^ were spread onto de Man–Rogosa–Sharpe (MRS) agar (HiMedia Laboratories LLC, Kennett Square, PA, USA). The plates were incubated at 37 °C for 48 h under anaerobic conditions using a 2.5 L anaerobic jar with a carbon dioxide-generating system (BD GasPak™ EZ, BD, Sparks, MD, USA), following the standard colony-count technique [21]. Colonies formed on MRS agar plates were used to determine the viable counts of probiotic bacteria. The results are expressed as logarithmic colony-forming units (log CFU/mL).

### 2.6. Extraction and Analysis of Short-Chain Fatty Acids (SCFAs)

SCFAs were extracted using a modified acidified organic solvent method according to Ribeiro et al. (2018) [22]. Briefly, 20 mg of fermented fecal samples were weighed into 1.5 mL microcentrifuge tubes and homogenized with 200 µL of distilled water using a metal spatula. Subsequently, 200 µL of an organic solvent mixture (n-butanol, tetrahydrofuran, and acetonitrile at a ratio of 50:30:20, *v*/*v*/*v*) was added, followed by 40 µL of 0.1 M HCl, 20 mg of citric acid, and 40 mg of NaCl. The mixture was vigorously vortexed for 1 min and centrifuged at 12,000 rpm at room temperature for 10 min. The resulting supernatant was collected into a new tube and stored at 4 °C prior to analysis. SCFA concentrations were analyzed by gas chromatography–mass spectrometry (GC–MS; Agilent 7250 GC-MS) at Eurofins Product Service (Thailand) Co., Ltd., Bangkok, Thailand.

### 2.7. Preliminary Investigation of KC Milk Tablets on Lipid Profiles and Cardiovascular Risk Markers in Hypercholesterolemic Subjects; Open-Label Pre–Post Assessment

#### 2.7.1. Study Design

An open-label trial with a 6-week intervention and pre–post analysis was conducted in adults with hypercholesterolemia. The study was performed in accordance with the principles of Good Clinical Practice (GCP) and complied fully with the ethical standards for clinical research as set forth in the Declaration of Helsinki. The study protocol was reviewed and approved by the full Institutional Review Board (IRB) of the Research Ethic Committee Panel 1, Faculty of Medicine, Chiang Mai University, Thailand (Approval no. 436/2025; Research ID: BIO-2568-0589). Written informed consent was obtained from all participants prior to study enrollment.

#### 2.7.2. Subjects and Interventions

Twenty Thai adults were recruited from the community surrounding Chiang Mai University through poster advertisements. Eligibility criteria included adults aged 30–65 years with hypercholesterolemia, defined by the presence of at least one of the following lipid abnormalities: serum total cholesterol (TC) ≥ 200 mg/dL, triglycerides (TG) ≥ 150 mg/dL, low-density lipoprotein cholesterol (LDL-C) ≥ 100 mg/dL, or high-density lipoprotein cholesterol (HDL-C) < 40 mg/dL, and who were not taking cholesterol-lowering medications. Exclusion criteria included a body mass index (BMI) > 35 kg/m^2^, smoking, being an athlete, diabetes mellitus, multiple allergies, gastrointestinal diseases, cancer, pregnancy, central nervous system or psychiatric disorders, traumatic injury, and the use of dietary supplements or other medications that could potentially affect cholesterol metabolism within 14 days prior to enrollment.

Participants’ demographic characteristics were recorded before and after the intervention. Physical activity levels were assessed using the Global Physical Activity Questionnaire (GPAQ), and energy intake was assessed using a 24 h dietary recall. Body fat percentage and basal metabolic rate (BMR) were measured using a body composition analyzer (Charder MA801, Charder Electronic Co., Ltd., Taichung, Taiwan).

For blood analysis, participants fasted overnight for 10–12 h prior to venous blood collection. Fasting plasma glucose, serum lipid profiles, inflammatory parameters, and key markers of cholesterol metabolism were assessed at baseline and after 6 weeks of intervention. All participants were instructed to maintain their usual dietary intake and physical activity patterns throughout the study period.

Eligible participants were instructed to consume 20 g of KC milk tablets once daily in the morning (between 08:00 and 12:00) for 6 weeks, either by chewing or swallowing them. During the intervention, participants were advised to avoid any additional dietary supplements. Compliance was assessed quantitatively by requiring participants to return empty sachets at each weekly contact. Returned sachets were counted by research staff to verify adherence to the prescribed daily consumption of 20 g KC milk tablets throughout the 6-week intervention period.

**Figure 1:**
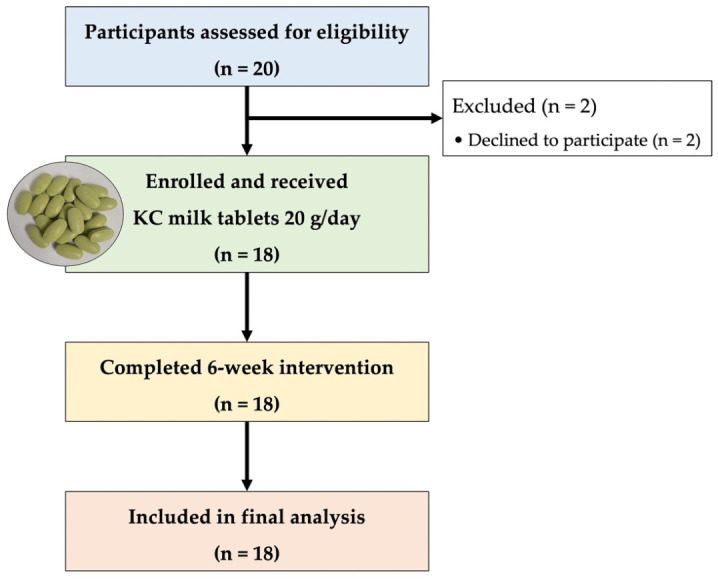
Flow diagram of participant recruitment, enrollment, intervention, and analysis. Twenty adults with hypercholesterolemia were assessed for eligibility. Two individuals declined to participate and were excluded (*n* = 1, discomfort during blood collection; *n* = 1, relocation of workplace). Eighteen eligible participants were enrolled and received KC milk tablets (20 g/day) for 6 weeks. All participants completed the intervention and were included in the final analysis.

To minimize potential confounding factors related to dietary intake, research staff contacted each participant weekly to encourage maintenance of their usual dietary habits throughout the study period. A schematic diagram of participant eligibility is shown in Figure 1.

Venous blood samples (10 mL) were collected by a registered nurse after an overnight fast of 10–12 h. Blood analyses were performed at baseline and at the end of the intervention period. A portion of the collected sample (8 mL of whole blood) was used for the analysis of fasting blood glucose and lipid profiles, including triglycerides, total cholesterol, high-density lipoprotein cholesterol (HDL-C), and low-density lipoprotein cholesterol (LDL-C), at the PROMPT Health Center, Faculty of Associated Medical Sciences, Chiang Mai University, Chiang Mai, Thailand.

The remaining 2 mL of blood was centrifuged at 10,000 rpm at 4 °C for 10 min. Serum samples were collected, aliquoted into vials, and stored at −80 °C until further analysis. Apolipoprotein E (ApoE), apolipoprotein B100 (ApoB100), 3-hydroxy-3-methylglutaryl-coenzyme A reductase (HMGCR), and tumor necrosis factor-alpha (TNF-α) were quantified using enzyme-linked immunosorbent assay (ELISA) kits with paired antibodies purchased from Wuhan Fine Biotech Co., Ltd. (Wuhan, Hubei, China).

Adverse events were monitored throughout the 6-week intervention period through weekly telephone contact with each participant. Participants were instructed to report any unusual symptoms or discomfort potentially associated with KC milk tablet consumption. Hematological safety was evaluated at baseline and post-intervention using complete blood count (CBC) and differential white blood cell counts (neutrophils, lymphocytes, monocytes, eosinophils, and basophils). Blood samples were analyzed at the PROMPT Health Center, Faculty of Associated Medical Sciences, Chiang Mai University, Chiang Mai, Thailand. All hematological parameters remained within standard laboratory reference ranges.

### 2.8. Statistical Analysis

The required sample size was estimated using STATA version 16 based on a one-sample paired *t*-test [23]. Based on this estimation, 20 subjects were enrolled to achieve 80% power at a significance level of 0.05, allowing for a 10% dropout rate.

Statistical analyses were performed using SPSS software (Version 22). Categorical variables are presented as frequencies and percentages, whereas continuous variables are presented as mean ± standard deviation (SD). The normality of difference scores (After − Before) was assessed using the Shapiro–Wilk test prior to analysis. Of the 16 variables examined, 14 were normally distributed and analyzed using paired *t*-tests, with LDL-C predefined as the primary outcome and the remaining 12 variables treated as secondary exploratory outcomes. Two variables, namely BMI (W = 0.869, *p* = 0.017) and Total Fat % (W = 0.832, *p* = 0.006), violated the normality assumption and were therefore analyzed using the Wilcoxon signed-rank test. A *p*-value < 0.05 was considered statistically significant.

To control for multiple comparisons, the Benjamini–Hochberg (BH) procedure was applied to control the false discovery rate (FDR) at 5%. Effect sizes were reported as Cohen’s d for paired *t*-tests or rank-biserial correlation for the Wilcoxon signed-rank test.

## 3. Results

### 3.1. Characteristics and Nutritional Values of KC Milk Tablets

Approximately 35% (*w*/*w*) of freeze-dried kale powder and hot-air-dried carrot powder, at a ratio of 1.25:1, were thoroughly blended with milk powder and other ingredients and subsequently compressed into tablets using a tablet press machine. Each tablet had an average weight of 1020 ± 5.35 mg and exhibited a hardness of 46.50 ± 1.07 N, indicating suitable mechanical strength for handling and consumption. The tablets were uniform in an oval shape and light green color, reflecting proper mixing and compression of the powdered ingredients.

Nutritional values, including protein, total fat, cholesterol, total carbohydrate, total dietary fiber, soluble dietary fiber, insoluble dietary fiber, total sugars, ash, and moisture content, were determined using standard methods. Caloric content was also calculated and expressed as kilocalories (kcal). The nutritional composition per 20 g and 100 g serving, as well as the analytical methods used, are presented in Table 1. A 20 g serving of KC milk tablets provides 80 kcal of energy, including 4 g protein, 3.50 g total fat, 3 mg cholesterol, 10 g total carbohydrates, 5.30 g dietary fiber (0.70 g soluble fiber and 4.60 g insoluble fiber), and 6 g total sugars.

### 3.2. Polyphenolic Composition and Antioxidant Properties of KC Milk Tablets

**Figure 2:**
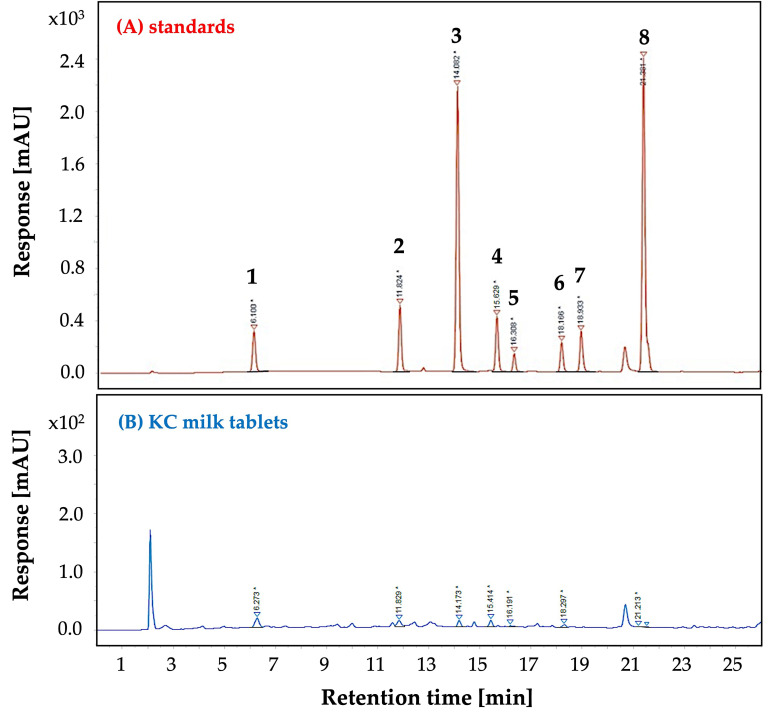
HPLC chromatograms of standards (**A**) and KC milk tablets (**B**). The x axis represents retention time, and AU on the y axis represents absorbance units (a signal corresponding to the response created by the detector) at 278 nm. Chromatogram of phenolic compounds: 1 = Gallic acid, 2 = Chlorogenic acid, 3 = Vanillin, 4 = *p*-Coumaric acid, 5 = Sinapic acid, 6 = Rutin, 7 = Quercetin, and 8 = Cinnamic acid. The symbol λ denotes peaks automatically identified by the HPLC software based on retention time matching.

Table 2 shows that the total phenolic and total flavonoid contents in KC milk tablets were 102.13 ± 0.087 mg gallic acid equivalents (GAE)/g DW and 0.76 ± 0.006 mg quercetin equivalents (QE)/g DW, respectively. Polyphenolic constituents, including gallic acid, chlorogenic acid, vanillin, *p*-coumaric acid, sinapic acid, rutin, and quercetin, were detected at concentrations of 2.71 ± 0.001, 0.11 ± 0.002, 0.23 ± 0.001, 0.04 ± 0.001, 0.37 ± 0.001, 0.22 ± 0.001, and 0.16 ± 0.002 mg/g, respectively. However, cinnamic acid was not detected in the analyzed samples. The representative HPLC chromatogram is shown in Figure 2.

The antioxidant capacity of KC milk tablets was evaluated by the DPPH, FRAP, and ABTS methods, with EC_50_ values of 0.18 ± 0.002 mg/mL, 0.45 ± 0.002 mg/mL, and 0.10 ± 0.012 mg/mL, respectively.

### 3.3. Prebiotic Potential and SCFA Production of KC Milk Tablets

The prebiotic potential of KC milk tablets was evaluated based on their ability to support the growth of microorganisms during *in vitro* fermentation. The results indicated that KC milk tablets effectively supported the growth of both *L. casei* and *B. longum*. An increase in microbial populations was observed during fermentation in the presence of KC milk tablets. Specifically, the colony density of *L. casei* increased from 7.86 ± 0.04 log CFU/mL to 11.21 ± 0.01 log CFU/mL, while *B. longum* increased from 10.58 ± 0.01 log CFU/mL to 12.90 ± 0.01 log CFU/mL after fermentation. The detailed microbial growth results are presented in Table 3.

SCFA production including acetic acid, propionic acid, and butyric acid, was analyzed after 48 h of fermentation with *L. casei* and *B. longum*. The concentrations of SCFAs before and after fermentation are summarized in Table 4. During fermentation with *L. casei*, the concentrations of acetic acid and propionic acid significantly increased after 48 h compared with the initial baseline (*p* value < 0.01), whereas butyric acid production did not show a significant change. In contrast, fermentation with *B. longum* resulted in a significant increase only in acetic acid concentration (*p* value < 0.05), while the levels of propionic acid and butyric acid remained statistically unchanged relative to baseline. Overall, these preliminary results suggest that KC milk tablets may possess prebiotic potential *in vitro*, as indicated by their capacity to support probiotic bacterial growth and stimulate microbial fermentation, leading to the production of SCFAs.

### 3.4. Effects of KC Milk Tablet Intervention on Cardiovascular Risk Factors in Hypercholesterolemic Subjects: An Open-Label Trial

#### 3.4.1. Participant Characteristics

Twenty subjects with hypercholesterolemia were initially enrolled (*n* = 20). Two participants withdrew prior to completion: one due to discomfort during venous blood collection and one due to relocation of workplace. Data from 18 subjects who completed the 6-week study period were included in the analysis. The final sample consisted of three males and fifteen females, with a mean age of 44.25 ± 7.11 years. After 6 weeks of intervention, no significant changes in BMI were observed. Physical activity and energy intake also remained unchanged between baseline and post-intervention (data are presented in Supplementary Table S1).

During the study, no adverse events related to KC milk tablet consumption were reported throughout the 6-week intervention period. Hematological parameters remained within normal reference ranges for all participants at baseline and post-intervention (Supplementary Table S2), with no clinically significant abnormalities observed.

#### 3.4.2. Effects of KC Milk Tablets on Cardiovascular Risk Factors in Hypercholesterolemic Subjects

Changes in cardiovascular risk factors after KC milk tablets consumption are presented in Table 5. After 6 weeks of daily consumption, subjects’ BMI, body fat percentage, and blood pressure did not significantly change compared with baseline values. However, subjects who consumed KC milk tablets showed a significant increase in basal metabolic rate (BMR) (*p* value = 0.033, 95% CI: −122.528 to −5.694). Fasting plasma biochemical parameters, including glucose, triglycerides, cholesterol, and the inflammatory marker (TNF-α), remained unchanged. Notably, LDL-C significantly decreased by 12.22%, whereas HDL-C increased by 18.38% (*p* value = 0.005, 95% CI: 4.873 to 23.771 and *p* value = 0.001, 95% CI: −13.335 to −4.998, respectively) after KC milk tablets consumption. Plasma ApoE and ApoB100 are important determinants of blood cholesterol levels and are associated with atherosclerosis development. Interestingly, subjects who consumed KC milk tablets daily showed a significant decrease in ApoB100 concentration (*p* value = 0.035, 95% CI: 0.739 to 18.723) and no significant changes in ApoE concentration compared with pre-consumption levels. In addition, our investigation focused on HMGCR, which is recognized as the key rate-limiting enzyme of the mevalonate pathway in cholesterol biosynthesis. Interestingly, circulating HMGCR levels were reduced after KC milk tablet consumption (*p* value = 0.004, 95% CI: 0.035 to 1.013) and it should be noted that this measurement does not directly reflect enzyme inhibition.

To address the issue of multiple comparisons, the BH procedure was applied to control the FDR. Following BH correction, HDL-C (adjusted *p* value = 0.015), LDL-C (adjusted *p* value = 0.038), and HMGCR (adjusted *p* value = 0.018) retained statistical significance, whereas BMR (adjusted *p* value = 0.124) and ApoB100 (adjusted *p* value = 0.107) were not significant after correction. The magnitude of intervention effects was further assessed using Cohen’s d, interpreted as small (d = 0.2), medium (d = 0.5), or large (d = 0.8) according to conventional criteria. HDL-C demonstrated a large effect size (d = −1.094), while LDL-C (d = 0.754), HMGCR (d = 0.765), BMR (d = −0.546), and ApoB100 (d = 0.522) demonstrated medium effect sizes, indicating moderate clinical relevance despite the small sample size.

## 4. Discussion

We developed novel milk tablets formulated from kale and carrot as a convenient functional food for daily consumption, providing dietary fiber and phenolic compounds with potential hypocholesterolemic activity. Milk tablets are gaining global popularity and are widely available in various flavors. However, their functional health benefits remain incompletely understood. In accordance with the Notification of the Ministry of Public Health (No. 352) B.E. 2556 (2013) of Thailand, the formulation was standardized to contain not less than 65% total milk solids. In our formulation, approximately 35% (*w*/*w*) of a combined kale and carrot powder was incorporated, enabling the product to meet the regulatory criteria for classification as a milk tablet and supporting its potential for commercial application. Moreover, the product provides 80 kcal per 20 g of daily serving and can be positioned as a low-calorie milk tablet. The KC milk tablets contained 5.30 g of dietary fiber per serving and were rich in polyphenolic compounds, with gallic acid identified as a major constituent.

The present study found preliminary data suggesting that KC milk tablets may possess prebiotic potential, based on enhanced growth of *L. casei* and *B. longum* observed in an in vitro fermentation model, alongside increased production of SCFAs, notably acetic and propionic acids. Dietary fiber has long been recognized as a prebiotic substrate capable of promoting beneficial bacterial growth and contributing to gut health, energy metabolism, and immune regulation [24,25,26]. The fermentation of dietary fiber by gut bacteria is well-established as a key driver of SCFA production, yielding acetate, propionate, and butyrate as primary metabolites [27]. Phenolic compounds have also been shown to support SCFAs generation through their own fermentative interactions with the microbiota [28]. Among these, gallic acid has drawn particular attention for its ability to enrich beneficial microbial populations and their associated metabolite outputs, with downstream benefits for gut health [29]. Taken together, the dietary fiber and gallic acid content of KC milk tablets may both play a role in driving SCFA production, a process linked to antihyperglycemic, hypolipidemic, and anti-inflammatory outcomes in prior research [30,31,32].

The health benefits, particularly the possible cholesterol-lowering effects of KC milk tablets, were preliminarily investigated in this study through an open-label, single-arm, pre–post-trial conducted in adults with hypercholesterolemia. In this exploratory study, daily consumption of KC milk tablets for 6 weeks was associated with increased HDL-C levels and reduced LDL-C concentrations, together with reductions in circulating ApoB100 and HMGCR levels following the intervention. On the other hand, TNF-α, ApoE, TG, and TC showed no significant changes. Similarly, a randomized, controlled, open-label, parallel-group study by Gulati et al. (2017) [33] demonstrated that 3 g of soluble fiber consumed for 4 weeks significantly reduced LDL-C levels. Other studies have also shown that dietary fiber from various food sources is associated with a reduced risk of cardiovascular disease by suppressing cholesterol levels [34,35].

Accumulating evidence suggests that SCFAs exert diverse physiological effects on lipid and cholesterol metabolism [27]. For example, acetate can act as a substrate for cholesterol synthesis and lipogenesis in peripheral tissues, whereas propionate has been reported to suppress hepatic cholesterol synthesis, thereby contributing to reduced circulating cholesterol levels [36]. In addition, propionate lower cholesterol concentrations by stimulating CYP7A1 activity, which enhances fecal bile acid excretion, and by inhibiting HMGCR activity [37,38]. In contrast, butyrate primarily functions as an energy source for intestinal epithelial cells and plays an important role in maintaining gut health through the regulation of energy balance, lipid metabolism, glucose homeostasis, and inflammatory responses [39]. Based on *in vitro* fermentation data, KC milk tablets demonstrated the capacity to support SCFA production, particularly acetic and propionic acids. It is therefore plausible, though not directly demonstrated in this study, that SCFA production may have contributed to the observed lipid changes, including the reductions in LDL-C and ApoB100 and the increase in HDL-C. Regarding circulating HMGCR levels, the observed reduction may reflect subsequent changes associated with altered cholesterol metabolism; however, it should be noted that this measurement reflects circulating HMGCR protein concentration as assessed by ELISA and does not constitute direct evidence of enzyme inhibition, reduced enzymatic activity, or suppression of hepatic cholesterol biosynthesis. Furthermore, since gut microbiota composition was not directly analyzed in study participants, any proposed link between in vitro SCFA production and the blood lipid profile outcomes remains preliminary. The observed changes in lipid parameters may also reflect other dietary or lifestyle factors, including hormonal fluctuations related to menopausal transition, that were not fully controlled given the open-label, uncontrolled study design.

Although the dietary fiber content of the product did not reach the level recommended according to a systematic review and meta-analysis for reducing CVD risk factors (25–30 g/day) [40], the preliminary findings of this study suggest that the presence of gallic acid may exert synergistic effects with dietary fiber to support lipid homeostasis. Similarly, previous studies have reported that gallic acid enhances cholesterol efflux, promotes bile acid metabolism, and regulates lipid homeostasis through signaling pathways, including AMPK and PPARα [41,42,43,44], collectively contributing to its lipid-lowering potential. Several studies have suggested that natural dietary fiber–phenolic conjugates may modulate the gut microbiota, leading to a range of beneficial physiological properties, including prebiotic, antioxidant, hypoglycemic, lipid-lowering, and anti-inflammatory activities [45,46,47]. Therefore, the combined presence of dietary fiber and gallic acid in KC milk tablets may be associated with improved lipid profiles. However, these findings require confirmation regarding the prevention or management of elevated cholesterol levels through well-designed randomized controlled trials.

Several important limitations of this study should be acknowledged. First, the in vitro fermentation experiment did not include an established positive prebiotic control such as inulin or fructooligosaccharides. The experimental design focused primarily on baseline screening, utilizing a negative control group without probiotic inoculation to assess endogenous or non-specific substrate alterations. The absence of a positive prebiotic control restricts our ability to perform a comparative efficacy analysis and limits the interpretation of the prebiotic activity of KC milk tablets relative to established prebiotics. Additionally, gut microbiota composition was not directly analyzed in study participants; therefore, the proposed link between in vitro SCFA production and the observed clinical lipid changes remains speculative and cannot be directly inferred from the current data. Future studies should include both a positive prebiotic control and direct experimental validation of SCFA production, as well as high-throughput gut microbiome sequencing to accurately profile the relative abundance of beneficial bacteria.

Second, limitations related to the study design and participant characteristics should be acknowledged. The open-label, single-arm, pre–post design without randomization or placebo control group, introduces a significant risk of bias led to a significant risk of bias, and therefore the observed changes cannot be definitively attributed to KC milk tablet consumption, as other confounding factors cannot be excluded. Furthermore, the predominantly female cohort, whose mean age of 44.25 years may overlap with the menopausal transition period, limits the reproducibility and generalizability of the findings across broader populations, as hormonal changes during this phase are known to modulate lipid metabolism [48,49]. In this regard, previous studies evaluating dietary supplements and functional foods for LDL-C reduction have included both male and female participants across a wider age range, demonstrating that mixed-sex study designs are both feasible and informative in this research context [50]. Future studies should therefore conduct a placebo-controlled randomized design with sex-stratified analysis and a more balanced sex distribution to better evaluate the cholesterol-lowering efficacy of KC milk tablets across diverse populations.

Third, an unexpected increase in BMR was observed following the intervention despite no significant changes in body weight, BMI, or body fat percentage. BMR was estimated using bioelectrical impedance analysis under standardized fasting conditions. It is plausible that SCFA-mediated signaling pathways, particularly through G-protein coupled receptor signaling pathways (FFAR2 and FFAR3), have been reported to modulate metabolic functions [51], and increased dietary fiber and polyphenol intake have been suggested to influence energy metabolism independently of body composition changes [52]. However, measurement variability inherent to BIA-based BMR estimation may also have contributed to this finding and may not directly reflect the effect of KC milk tablet consumption. The physiological relevance of this observation therefore remains unclear and requires further investigation using more precise metabolic measurement techniques such as indirect calorimetry.

Fourth, as shown in Supplementary Table S1, caloric intake appeared to increase following the intervention; however, this change did not reach statistical significance and no significant changes in body weight or BMI were observed, suggesting that the dietary changes may not have substantially influenced the observed lipid outcomes. This may partly reflect the limitations of self-reported 24 h dietary recall in capturing actual dietary behavior, and unmeasured changes in dietary quality or macronutrient composition could have contributed to the observed lipid outcomes. Future studies should consider using more comprehensive dietary assessment tools, such as multiple-day food records or objective biomarkers of dietary intake, to better control for dietary confounding and strengthen the validity of the findings.

Regarding safety, no adverse events related to KC milk tablet consumption were reported throughout the 6-week intervention period, and hematological parameters including CBC and WBC differential remained within normal reference ranges for all participants, suggesting that KC milk tablets may be safe for short-term consumption at the dose used in this study. However, liver and renal function tests were not performed in this study, which limits the completeness of the safety assessment. Future studies should incorporate a full safety panel including liver function tests and renal function panels to more comprehensively evaluate the safety profile of KC milk tablets, particularly with longer-term consumption.

In summary, this preliminary study suggests that daily consumption of KC milk tablets may be associated with improvements in lipid profiles, particularly increased HDL-C and reduced LDL-C, among individuals with hypercholesterolemia. Supporting evidence from in vitro fermentation further points to the potential prebiotic properties of the product, reflected by enhanced growth of *L. casei* and *B. longum* alongside increased SCFA production. Nevertheless, the open-label, single-arm design and lack of a placebo group suggest that these findings should be taken as preliminary. Future research including gut microbiota sequencing, placebo-controlled randomized trials with sex-stratified analysis, and longer follow-up periods will be essential in establishing the potential effect of KC milk tablets for cholesterol management.

## 5. Conclusions

**Figure 3:**
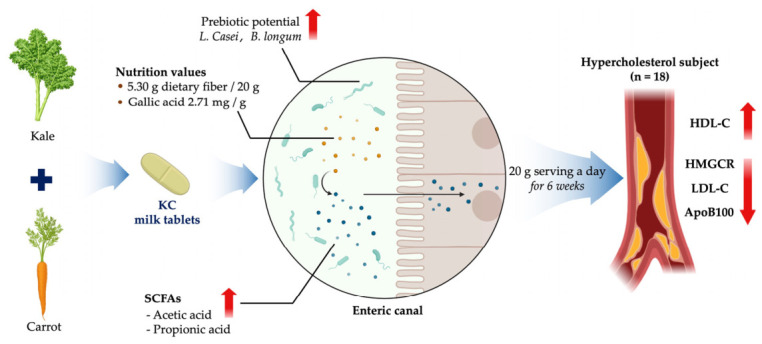
Proposed mechanism of the cholesterol-related effects of KC milk tablets. KC milk tablets containing dietary fiber and phenolic compounds from kale and carrot demonstrated prebiotic potential and enhanced SCFA production in vitro. In hypercholesterolemic subjects, daily consumption of 20 g for 6 weeks was associated with increased HDL-C levels and reduced circulating LDL-C, ApoB100, and HMGCR levels.

KC milk tablets, derived from natural vegetable sources including kale and carrot, show preliminary potential associated with cholesterol metabolism, in which the reduction in circulating HMGCR levels may be partly responsible for the decreased LDL-C and ApoB100 and increased HDL-C levels. These effects observed in this study may be attributed to their dietary fiber content and phenolic compounds such as gallic acid found in KC milk tablets as shown in Figure 3.

Therefore, daily consumption of 20 g of KC milk tablets for 6 weeks may help maintain healthy cholesterol levels and serve as a convenient and healthy snack option. However, further studies are needed to confirm these effects, particularly regarding efficacy through randomized controlled trials.

## 6. Patents

A patent application related to this work is currently under submission.

## Figures and Tables

**Table 1:** Nutritional values of KC milk tablets.

Test Item	Results/20 g	Results/100 g	Methods
Energy (kcal)	80	422.76	Method of Analysis for Nutrition Labeling (1993), Chapter 6 Proximate and Mineral Analysis.
Protein (g)	4	20.97	AOAC (2019) 991.20.
Total fat (g)	3.50	17.60	AOAC (2019) 985.29.
Cholesterol (mg)	3	14.64	In house method based on Compendium of methods for Food Analysis (2003).
Total carbohydrate (g)	10	51.77	Method of Analysis for Nutrition Labeling (1993), Chapter 6 Proximate and Mineral Analysis.
Total dietary fiber (g)	5.30	26.52	AOAC (2019) 985.29.
Soluble dietary fiber (g)	0.70	3.37	AOAC (2019) 991.42.
Insoluble dietary fiber (g)	4.60	23.15	AOAC (2019) 993.19.
Total sugars (g)	6	31.69	In-house method based on compendium of method for food analysis (2003).
Ash	-	5.64	AOAC (2019) 920.153.
Moisture	-	4.02	AOAC (2019) 950.46 (B).

**Table 2:** Polyphenolic composition and antioxidant properties of KC milk tablets. Values are expressed as mean ± SD (*n* = 3). EC_50_ = half-maximal effective concentration; GAE = gallic acid equivalents, GAE = gallic equivalent.

Polyphenolic Compounds	KC Milk Tablets
Total phenolic content (mg GAE/g DW)	102.13 ± 0.087
Total flavonoid content (mg Quercetin/g DW)	0.76 ± 0.006
Gallic acid (mg/g DW)	2.71 ± 0.001
Chlorogenic acid (mg/g DW)	0.11 ± 0.002
Vanillin (mg/g DW)	0.23 ± 0.001
*p*-Coumaric acid (mg/g DW)	0.04 ± 0.001
Sinapic acid (mg/g DW)	0.37 ± 0.001
Rutin (mg/g DW)	0.22 ± 0.001
Quercetin (mg/g DW)	0.16 ± 0.002
Cinnamic acid (mg/g DW)	ND
Antioxidant properties	
Scavenging ability of DPPH radicals (EC_50_; mg/mL)	0.18 ± 0.002
FRAP assay (EC_50_; mg/mL)	0.45 ± 0.002
Scavenging Ability of ABTS radicals (EC_50_; mg/mL)	0.10 ± 0.012

**Table 3:** Growth of *L. casei* and *B. longum* in response to fermentation with KC milk tablets. Data are presented as Log CFU/mL. Results are mean  ±  standard deviation (SD).

Tests	At 0 Hour		At 48 Hours	
	pH Value	Colony Count (Log CFU/mL)	pH Value	Colony Count (Log CFU/mL)
KC milk tablet	6.61 ± 0.12	No growth	6.01 ± 0.39	No growth
KC milk tablet *+ L. casei*	6.41 ± 0.01	7.86 ± 0.04	5.34 ± 0.01	11.21 ± 0.01
KC milk tablet *+ B. longum*	6.37 ± 0.06	10.58 ± 0.01	5.19 ± 0.06	12.90 ± 0.01

**Table 4:** SCFA contents following in vitro fermentation of KC milk tablets with *L. casei* or *B. longum*. Results are mean  ±  standard deviation (SD). * *p* value on the paired *t*-test at *p* < 0.05, ** *p* value on the paired *t*-test at *p* < 0.01.

SCFA Production	KC Milk Tablet *+ L. casei*		KC Milk Tablet *+ B. longum*	
	Pre-Fermentation	Post-Fermentation	Pre-Fermentation	Post-Fermentation
Acetic Acid (mg/L)	86.02 ± 22.83	160.57 ± 31.63 *	105.21 ± 56.95	457.27 ± 83.56 *
Propionic Acid (mg/L)	13.70 ± 1.32	17.58 ± 1.16 **	18.40 ± 8.73	11.49 ± 2.97
Butyric Acid (mg/L)	11.42 ± 2.30	16.53 ± 8.89	11.95 ± 4.32	11.68 ± 3.68

**Table 5:** Effects of KC milk tablets on lipid profiles and cardiovascular risk factors in hypercholesterolemic subjects before and after intervention. Results are mean  ±  standard deviation (SD). * *p* value on the paired *t*-test at *p* < 0.05, ** *p* value on the paired *t*-test at *p* < 0.01, *** *p* value on the paired *t*-test at *p* < 0.001.

Parameters	Before	After	95% Confidence Interval (CI)		*p* Value (Pre–Post Analysis)	BH Adjusted *p* Value	Cohen’s d
			Lower	Upper			
Sex	F = 15 M = 3						
Age	44.25 ± 7.11						
Systolic Blood Pressure (mmHg)	120.85 ± 16.52	123.78 ± 13.32	−10.147	2.703	0.238	0.595	−0.288
Diastolic Blood Pressure (mmHg)	78.20 ± 7.65	78.89 ± 8.67	−6.299	4.299	0.695	0.802	−0.094
Body Mass Index	25.85 ± 4.56	25.35 ± 3.83	−0.998	1.953	0.504	1	0.161
Body Fat Percentage (%)	34.62 ± 7.15	31.53 ± 9.31	−2.627	7.416	0.327	0.614	0.245
Basal Metabolic Rate (kcal/day)	1259.80 ± 244.82	1306.06 ± 215.47 *	−122.528	−5.694	0.033	0.124	−0.546
Fasting Blood Sugar (mg/dL)	81.89 ± 7.96	81.72 ± 8.44	−4.334	4.667	0.939	0.939	0.018
Cholesterol (mg/dL)	191.83 ± 29.83	187.78 ± 26.96	−6.425	14.536	0.426	0.71	0.192
Triglyceride (mg/dL)	123.61 ± 62.40	129.11 ± 67.96	−41.416	30.416	0.751	0.804	−0.074
HDL-C (mg/dL)	49.89 ± 8.82	59.06 ± 8.73 ***	−13.335	−4.998	0.001	0.015	−1.094
LDL-C (mg/dL)	117.22 ± 23.05	102.90 ± 21.67 **	4.873	23.771	0.005	0.038	0.754
Apolipoprotein E (ng/mL)	37.59 ± 20.77	44.74 ± 22.40	−24.165	6.571	0.245	0.614	−0.276
Apolipoprotein B100 (ng/mL)	39.84 ± 16.59	30.11 ± 16.59 *	0.739	18.723	0.035	0.107	0.522
HMG-CoA reductase (ng/mL)	1.96 ± 1.06	1.42 ± 0.50 **	0.190	0.885	0.004	0.018	0.765
TNF-α (pg/mL)	244.33 ± 116.07	248.27 ± 87.00	−0.248	0.452	0.546	1	−0.029

## Data Availability

The authors confirm that the data supporting the findings of this study are available within the article and its Supplementary Materials. Raw data that support the findings of this study are available upon reasonable request.

## Supplementary Materials

The following supporting information can be downloaded at: https://www.mdpi.com/article/10.3390/foods15122235/s1, Table S1: Physical activity and energy intake of participants before and after KC milk tablet consumption. Table S2: Hematological parameters of participants before and after KC milk tablet consumption. Figure S1: Individual changes in HDL-C concentrations during the intervention period. Each solid line represents an individual participant. The dashed line represents the mean ± SD of the study population at each time point. Values were measured at baseline and after 6 weeks of KC milk tablet supplementation. Figure S2: Individual changes in LDL-C concentrations during the intervention period. Each solid line represents an individual participant. The dashed line represents the mean ± SD of the study population at each time point. Values were measured at baseline and after 6 weeks of KC milk tablet supplementation. Figure S3: Individual changes in HMG-CoA reductase levels during the intervention period. Each solid line represents an individual participant. The dashed line represents the mean ± SD of the study population at each time point. Values were measured at baseline and after 6 weeks of KC milk tablet supplementation.

## Abbreviations

The following abbreviations are used in this manuscript:

ApoB100	Apolipoprotein B100
ApoE	Apolipoprotein E
CFU	Colony Forming Unit
CYP7A1	Cholesterol 7 alpha-hydroxylase
EC_50_	Half Maximal Effective Concentration
ELISA	Enzyme-linked immunosorbent assay
HDL-C	High-Density Lipoprotein Cholesterol
HMGCR	3-Hydroxy-3-methylglutaryl-coenzyme A reductase
LDL-C	Low-Density Lipoprotein Cholesterol
TNF-α	Tumor Necrosis Factor-alpha
SCFAs	Short-chain fatty acids
