# Supplementary material for: Dietary Fiber–Phenolic Milk Tablets Are Associated with Improved Lipid Profiles and Reduced Circulating HMGCR Levels in Hypercholesterolemic Subjects: An Open-Label Pre–Post Study
Source: Foods. 2026 Jun 21;15(12):2235. doi: 10.3390/foods15122235 (PMC13297886; doi:10.3390/foods15122235)
Supplement: Supplementary file 1 [file foods-15-02235-s001.zip › foods-4328110-supplementary.pdf]

Table S1: Physical activity and energy intake of participants before and after KC milk tablet consumption.

GPAQ categorical questions	Before		After	
	n	%	n	%
Vigorous-intensity activity at work	1	5.56	1	5.56
Moderate-intensity activity at work	13	72.22	14	77.78
Sedentary at work	4	22.22	3	16.67
Activity during travel	1	5.56	1	5.56
Vigorous-intensity activity in leisure time	2	11.11	1	5.56
Moderate-intensity activity in leisure time	8	44.44	9	50
Sedentary in leisure time	8	44.44	7	44.44
Total physical activity MET-min (Mean ± SD)	1828.52 ± 1,061.90		2021.6 ± 18	
Energy intake from 24-hour dietary recall (kcal/day)	2235 ± 405.13		2415 ± 355.74	

Table S2: Hematological parameters of participants before and after KC milk tablet consumption.

Hematological parameters	Classified reference range	Unit	Before			After		
WBC	5.3-8.5	10^3 Cells/ µl	7.17	±	1.01	7.17	±	0.83
RBC	3.9-5.00	10^6 Cells/ µl	4.89	±	0.46	4.81	±	0.41
HGB	12.5 - 14.3	g/dl	13.04	±	1.42	12.95	±	1.34
HCT	37.1 - 42.7	%	39.19	±	3.39	38.44	±	3.40
MCV	82 - 97	fL	82.6	±	7.95	84.17	±	5.64
MCH	27 - 31	pg	28.0	±	2.78	28.38	±	2.80
MCHC	32 - 36	g/dl	33.19	±	1.37	33.66	±	1.29
RDW-CV	11.7 - 15	%	13.69	±	1.58	13.16	±	0.83
Platelete count	157 - 399	10^3/ µl	309.55	±	56.42	300.44	±	64.19
Neutrophil	49 - 69	%	56.95	±	5.60	56.17	±	5.45
Lymphocyte	24 - 40	%	32.40	±	5.20	32.39	±	4.75
Monocyte	4-8	%	7.05	±	1.10	7.28	±	0.89
Eosinophil	0 - 3	%	1.85	±	0.81	1.78	±	0.81
Basophil	0 - 1	%	0.20	±	0.41	0.39	±	0.50

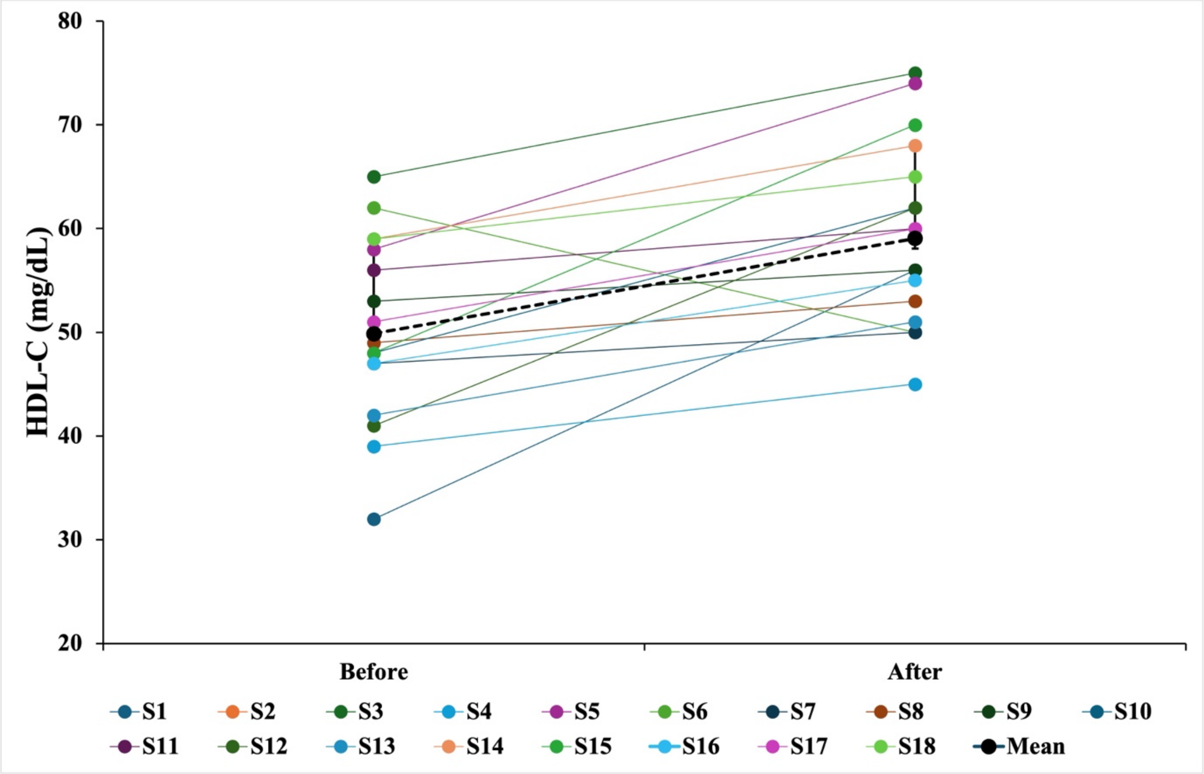

Figure S1: Individual changes in HDL-C concentrations during the intervention period. Each solid line represents an individual participant. The dashed line represents the mean± SD of the study population at each time point. Values were measured at baseline and after 6 weeks of KC milk tablet supplementation.

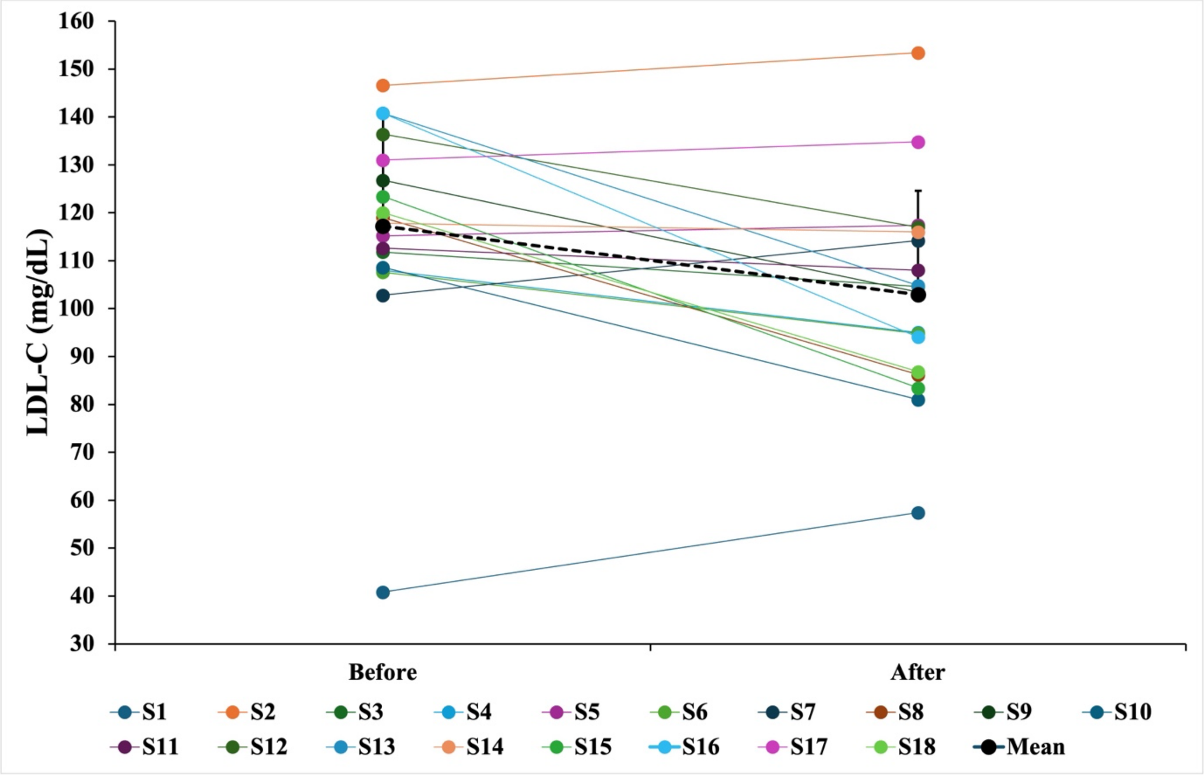

Figure S2: Individual changes in LDL-C concentrations during the intervention period. Each solid line represents an individual participant. The dashed line represents the mean±SD of the study population at each time point. Values were measured at baseline and after 6 weeks of KC milk tablet supplementation.

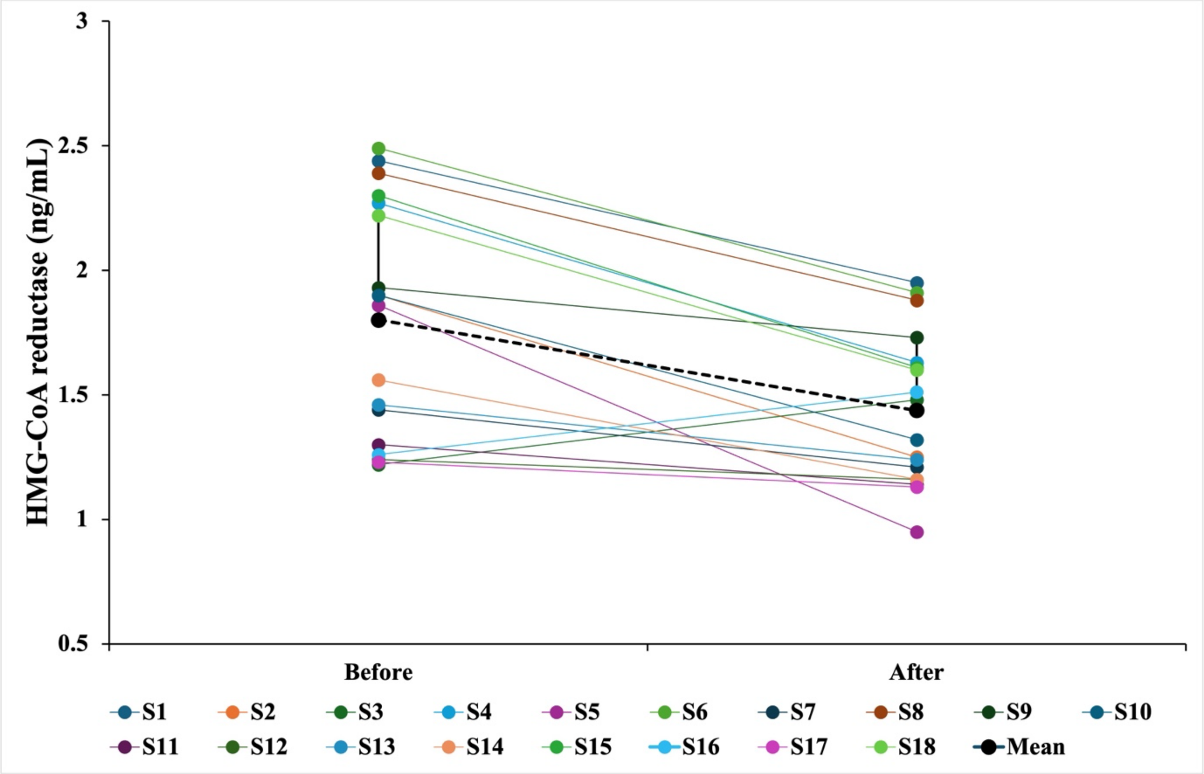

Figure S3: Individual changes in HMG-CoA reductase levels during the intervention period. Each solid line represents an individual participant. The dashed line represents the mean±SD of the study population at each time point. Values were measured at baseline and after 6 weeks of KC milk tablet supplementation.
